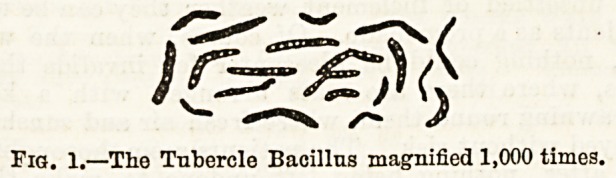# The Hospital Nursing Supplement

**Published:** 1894-09-22

**Authors:** 


### "The Hospital" Nursing Mirror.

Being the Extra Nursing Supplement of "The Hospital" Newspaper.

[Contributions for this Supplement should be addressed to the Editor, The Hospital, 428, Strand, London, W.C., and should have the word "Nursing" plainly written in left-hand top corner of the envelope.]

### News from the Nursing World.

### ROYAL NATIONAL PENSION FUND FOR NURSES.

We are informed that the offices of this Fund are to be at 28, Finsbury Pavement, London, on and after September 29th. The new premises, which appear to be very convenient and commodious, are about five minutes' walk from the present address, on the first floor, above Milner's Safe Company's office. The entrance is in West Street, which runs from Finsbury Pavement into Finsbury Circus. For North, North-West, and East London the new offices are most conveniently situated, being close to Liverpool and Broad Street Stations, and within a stone's throw of the tram lines which terminate at Finsbury Square. Being near to Moorgate Street Station, nurses coming from all parts of London will experience little difficulty, while all the 'buses running from the Western districts pass through London Wall.

### OUR CHRISTMAS COMPETITIONS.

We hope our numerous readers are bearing our Christmas work competitions in mind. It is most desirable that a good supply of warm garments should be forthcoming this winter. We get more demands each year for "just one parcel" to help to make the sick who spend Christmas in hospital look forward hopefully to the convalescence which will be eased by "something warm" to wear when leaving the comfortable ward. The prizes offered are: 20s. for the most serviceable dressing gown; 10s. for the best flannel shirt; 7s. 6d. for the best flannel petticoat; 7s. 6d. for the best over-petticoat; 7s. 6d. for the best bed jacket; 5s. for the best knitted pair of men's socks; and 2s. 6d. for the second best pair.

### EPILEPTIC COLONIES.

A correspondent thinks that our recent paragraph on Bielefeld is inaccurate, but our remark that we have no institution in England like that colony of 1,300 inhabitants is correct. The Meath Home, founded after a visit to the German colony by Lady Meath, was opened in August, 1892, and accommodates fifty or sixty patients. The list of applicants being now a long one, a number of suitable cases have to wait a long time for admission. The best results are reported as following on the training and varied occupations supplied to the inmates, and the work so well begun now only needs increasing and developing to meet the urgent needs of the epileptics hitherto debarred from many advantages offered at Godalming.

### A NURSE-HEROINE.

The Workhouse Infirmary Nursing Association may certainly congratulate itself on the qualities of the nurse sent down to Newton Abbot. No doubt she required and received from time to time encouragement to carry on her brave crusade. Our readers will remember that a subscription was set on foot to pay the expenses attendant on the inquiry which was the result of Nurse Hinton's revelations. The sum raised amounted to £106, and, after defraying the expenses, a gold watch and a purse of £66 were presented to the nurse on September 11th. The watch is engraved with the following inscription: "Presented to Nurse Hinton, with a purse of sovereigns, in recognition of her humanity and courage in assisting to remedy the abuses in the Newton Abbot Workhouse."

### UNAVOIDABLE POSTPONEMENT.

The conviction of Her Royal Highness Princess Christian that the R.B.N. Association is "not ripe for a congress" has resulted in the definite postponement of the scheme of some of the members to attempt an International Congress of Nurses during the coming year. The promoters have, therefore, been constrained to acquiesce in the prudent decision of their president, to relegate the convention of a congress to "some future time." Princess Christian has graciously consented to hold a stall at the bazaar which is to take place in December, in aid of the funds of the Royal British Nurses' Association.

### THE WARD THERMOMETER.

"Where's the thermometer?" asks the nurse in charge of the ward. "Oh! I've given it to the new patient," responds her subordinate. "But why did you do that? You were told to take his temperature?" It needs prolonged experience to discriminate between the two proceedings, yet without such care it is impossible to eliminate such impostors as the man recently reported as having attracted attention in many hospital wards by his abnormal register. Whether a nurse took the temperature in all cases, or if the thermometer was merely given to the patient, will perhaps never be known; but doubtless it was in the former case that the imposture was detected and made public.

### PAUPERS AT PORTSEA.

A recent visit of two Local Government Board Inspectors to Portsea Island Workhouse has shown that the sick wards containing 350 patients are in the sole charge of the hospital matron by day, and without any nurse at all at night. Miss Court (the matron) is a trained nurse, and her devotion, and that of Dr. Knott, the medical officer, to their patients was most favourably commented on by the Board; but it is obviously impossible that 350 sick and infirm persons should receive adequate nursing from one pair of hands, and the "critical case of amputation" left in the care of an octogenarian inmate is doubtless only one amongst many unfortunate necessities. The inspectors report that they are satisfied that payments have been made to pauper attendants by the patients, naturally with detrimental results to other poor creatures who are without means. Some of the food is kept in the ward, slung in canvas bags under the tables, an arrangement which can hardly be commended.

### QUEEN'S NURSES AT PERTH.

Miss Græme, lady superintendent, and Miss M'Queen, assistant nurse, of the Perth Sick Poor Nursing Society, recently received their badges and brassards as Queen's nurses at the hands of the Dowager Duchess of Athole. They have both done excellent work in Perth, where their services are greatly appreciated by their poor countrymen.

### NURSING AT NEWTOWNARDS.

The first annual meeting of the Newtownards Society for Nursing the Sick Poor was held on the 3rd inst. in the Assembly Rooms, and it was a most satisfactory one. The society was inaugurated last year by Lady Londonderry, and the scheme has been carried out with marked success, the labours of the trained district nurse being fully appreciated by the patients and admirably supported by the ladies of the neighbourhood.

### TRAINING OF MIDWIVES IN INDIA.

At the Lahore Medical College the lady students who are candidates for the diploma in midwifery must be over twenty and under thirty-five years of age, able to read and write English. They must produce evidence of having studied midwifery under a recognised teacher for not less than eighteen months, and of having attended not less than twenty-four cases of labour. They are required to undergo an examination (written and oral) in midwifery up to the standard of Dr. Fancourt Barnes' "Manual for Midwives." There is a training home for nurses and midwives in connection with the Dufferin Maternity Hospital, Rangoon, where the course of instruction extends over one year, and candidates for admission must be between eighteen and thirty.

### JUSTICE TO CHILDREN.

All readers of "The Mirror" take an active interest in the children. The report of the National Society for the Prevention of Cruelty to Children, just issued, contains a ten years' review of its work. It shows that among the children dealt with 8,618 were known to be insured for a total sum of £44,210, and that 272 of these cases ended in death. These figures open up a wide field of possibilities, full of horrors to the intelligent and thoughtful. It further appears that 14,324 of the cases dealt with were found to be true, of which 10,457 were warned. 1,628 persons were convicted, who were condemned to pay £579 in fines, and had to undergo collectively terms of imprisonment which represent a total of upwards of 296 years. It is claimed that the punishments inflicted resulted in the creation of happier homes. The prison-ward division of families has on the whole and in the long run proved their unity, and has secured for the children better food, clothing, and reasonable treatment. The prolonged depression has seriously impeded the work of the society, the accounts showing unpaid liabilities amounting to nearly £6,000, although the whole of the reserved fund has been used during the past year. We are confident these facts have only to be widely known to secure a much more adequate response to the society's appeals in the future.

### EDUCATED NURSES.

As a rule, Poor Law Guardians have not laid themselves open to the accusation of demanding too high a standard of education from the nursing classes. Nevertheless, a Whitechapel Guardian did recently express himself not satisfied with the general education of the nurses employed by the Board. One of nurses occupied in the locality, therefore, has published a protest against the desire for a higher educational standard for nurses. An intelligent knowledge of reading and writing and a fair amount of common sense is, she argues, all that nurses are led to expect will be the mental qualifications required of them. She lays more stress on the need of moral attributes, such as gentleness, patience, neatness, unselfishness, and firmness. In this we quite agree with her. In the careful technical training of the nurse of to-day there is, especially in institutional work, too little importance attached to a nurse's fitness for her office of tending the sick, but a knowledge of reading and writing can hardly now be considered as a fair educational standard.

### LEPERS IN SOUTH AFRICA.

An inquiry has been held into the recent rioting of the lepers at Robben Island, and very sad descriptions are given of the unfortunate creatures' entreaties to the soldiers to put an end to their sufferings.

### AFTER THE EVENT.

Very wonderful productions are some of those deposited in the editorial letter-box, and they are most of them interesting in one way or another. Perhaps the most depressing communications are those which appeal for advice or an opinion as to the prudence of a step already taken by the writer. A woman old enough to have passed through her full training as a nurse ought to have more sense than to announce that, having entered into an engagement, she wants to know whether it's a wise one! If she cannot trust her own judgment she should certainly not rashly act on it. and it is obvious folly to wait to ask advice until after an irretrievable step has been taken. The letters which bear insufficient addresses and signatures are soon disposed of in the waste paper basket, but there still remains a large balance of interesting communications behind. It would be pleasant if more of our nurse friends would write us a few additional letters for publication. There are many general nursing subjects, holidays, and other experiences which, if condensed into the reasonable dimensions of a letter, would give pleasure to many sister readers. "Nurse, London," is the signature adopted by one of our latest correspondents, and she expresses surprise that her letters get no answers!

### SHORT ITEMS.

Nurse Fleming has succeeded to Nurse Rutherford's post at the Blairgowrie and Rattray Nursing Association. — The Executive Committee of the Middlesbrough Nursing Association states that there is every hope of the new premises connected with the Nurses' Home being opened early in October.—The Grimsby and District Nursing Association report 419 gratuitous visits paid during August.—Only two applications were received for the position of nurse at Castleton Union. One candidate was disqualified on account of her failing to appear before the Board, whilst the condition of health of the other applicant was a drawback. The Guardians decided to advertise again.—A recreation ground for the patients at the Hope Hospital, Eccles, was recently opened by Mr. D. Williams, the Chairman of Salford Union Infirmary Committee. He was presented with a silver gift key by the members of the committee. The grounds are very prettily laid out.—Miss Cullip, sister in charge of the Cheshunt Hospital, has been engaged by the Technical Education Committee for another course of the lectures on Home Nursing which she gave so successfully at Cheshunt last year.

### Diet in Disease.

By Mrs. Ernest Hart.

Series II.

### III.—CONSUMPTION.

Consumption was once thought to be both an incurable and an unpreventable disease. It came mysteriously; it often attacked the youngest and fairest; it destroyed promising careers, and unless it could be checked by the exile of the patient to a warmer climate than England, its cure was thought to be hopeless and death sooner or later [inevitable. Of late years some considerable progress has been made in respect both to its prevention and to its successful treatment. The disease has by the researches of Koch been shown to be due to the ravages of a minute bacillus in the tissues. The effort of medicine in the treatment of consumption is first to make the tissues resistent, and next to cut short the life of the bacilli or to limit their power for evil. This end is mainly accomplished by two means—giving the patient sunlight and fresh air, and regulating the diet, and next by the application of antisepsis.

**The Tubercle Bacillus.**—Before proceeding to tell how these objects can be attempted, I will describe the appearances, and life history of the tubercle bacillus. If a beam of sunlight fall through a chink of a shutter into a darkened room, a number of motes will be seen to be floating about in it. These motes made visible by the strong light are present everywhere in the atmosphere in countless numbers. If they be allowed to settle and are examined under a microscope, it will be seen that they are composed of particles of dust and of minute rod-like bodies or bacilli. These [bacilli, though simple in structure and closely resembling one another in shape, are yet so dissimilar in their action and life that they require certain soils and certain conditions in order to grow and multiply. It is probable that the tubercle bacillus which is pictured in Figure 1 is very largely distributed in the atmosphere, and if taken in with the breath it settles on and grows in the lungs, and produces phthisis or consumption; if it is taken with the food and it settles and grows in the intestines it produces consumption of the bowels; if it is introduced into the brain it causes tubercular meningitis, and if it finds its way into the marrow of the bones it causes abscesses of those bones. In order, however, to grow in the human body the tubercle bacillus must find the right soil, exactly in the same way as a seed will not sprout unless it falls on the right soil. The conditions which produce in the tissues of the lungs or elsewhere the nidus or soil proper for the growth and development of the tubercle bacillus are probably in some measure hereditary, and also largely due to the environment. The rebreathing of expired air, bad (damp) conditions of soil, and defective nutrition may be set down as predisposing causes. Why one person should contract consumption and another not is not fully known. Once, however, the tubercle bacillus is established in the tissues or the air vessels of the lungs it undergoes rapid multiplication. By its irritating presence inflammation is set up, the tissue breaks down into pus, and cavities are ultimately formed in the lung. The patient becomes emaciated, loses strength, and finally dies from exhaustion or from insufficient aëration of the blood, owing to the fact that a large part of the lung has broken down and has been spat up in coughing.

Fig. 1.—The Tubercle Bacillus magnified 1,000 times.

The **aims of treatment** in pulmonary consumption are to improve the health and render "the soil" for the bacillus more resistent, and to fight and conquer the bacillus, to deprive it of its proper nutriment, and, if one is not able to kill it outright, to render it weak and powerless to work mischief. It is, unfortunately, not yet known positively what substances are destructive of the life of the tubercle bacillus, but there is some reason to believe that oil and soda-salts are antagonistic to its life. Hence probably the well-known benefits which result from the use of cod-liver oil. How the oil acts is by no means clear. The opinion of Hughes Bennett, by whom its use was introduced, was that it prolonged life by improving the nutrition of the tissues. It is suggested now by others that the oil, by being burnt up in the body, absorbs the oxygen required for the active multiplication of the micro-organisms. But what we do know is, that if wasting can be checked, and the weight of the patient increased, the tubercle bacillus is often successfully combatted.

**Treatment by Super-Alimentation..**—A consumptive patient should be carefully weighed at frequent and regular intervals; if he gains weight it is well, but if he loses a serious effort must be made to induce him to take more food. Sometimes, if the fever is high and continuous, appetite is destroyed, and there is even a distaste for food. In such cases many French physicians following Dr. Débove, of Paris, recommend forced feeding, and the introduction of food into the stomach by means of the æsophegeal tube. They report that under this treatment the patient recovers appetite, rapidly gains in weight, his strength increases, and the cough, expectoration, and night sweats disappear. Without resorting, however, to these heroic methods, a patient can with advanbe "over-fed" in the normal way. Care should be taken to give him as much of fatty foods as he can possibly digest, and far more than enter into the usual dietary. Bread and butter, cream, cocoa, chocolate, and milk are all excellent foods for a consumptive, as well as the usual articles of a healthy dietary. Where cream is not well borne it may be rendered more digestible by adding to each wine-glassfull a teaspoonful of brandy, kirsch, or rum, with or without hot water. Milk may be rendered more digestible by adding to each tumblerful two tablespoonsful of hot water, to which about six grains of bi-carbonate of soda and five grains of common salt have been added. Malt extract is also very useful in facilitating the digestion of farinaceous foods.

**The Treatment by Koumiss.**—Koumiss is fermented mare's milk. It has long been a favourite beverage with the Tartars and other Asiatic tribes. In Russia consumptives go to certain stations on the Caspian Sea to undergo the koumiss cure. The secret of the whole thing is that koumiss is milk slightly fermented, and consequently highly digestible, and of which, therefore, large quantities can be taken without producing dyspepsia. The Russian mode of cure is to rise early and to take a glass of koumiss every half-hour, with the exception of two hours preceding dinner and supper. Meat and fats form the chief part of the meals; sweets, fruits, and salads are forbidden, as well as ices, coffee, and spirits. Koumiss is made in Europe from cow's milk, and it is particularly appropriate in cases where the temperature is high and the appetite impaired.

**The Treatment by Powdered Raw Meat.**—An excess of food can be given a consumptive more easily by administering powdered raw meat than by any other method. Dujardin Beaumetz, who is an advocate of this method of treatment, recommends that the powder should be prepared from the lean of beef, which is cut into small pieces and dried in a water bath. When thoroughly dried it is reduced to powder in a coffee mill. The powder may be be taken either with lentil flour in the form of soup or with milk, or rum punch. In this way an amount of powdered raw meat can be taken daily, representing several pounds of meat. Abundant food would be, however, of little use if not combined with an abundance of fresh air. The aseptic stimulating air of the mountains, as at Davos, the ozone and revivifying breezes of the ocean, the sunlight and warmth of the South, Torquay, the Riviera, and Orotava, are all invaluable in the treatment of consumption ; in fact, in some cases warmth, sunlight, and fresh air the aseptic atmosphere of high altitudes are sufficient to arrest the tubercular inflammation and to effect a cure. This result is probably due to the fact that increased vitality of the patient induced by placing him under healthful conditions resists the destructive action of the microbes.

**Tubercle Bacillus Conveyed by Milk.**—There is no doubt that the tubercle bacillus can be conveyed to the human subject by milk from tuberculous cows, and that children have been infected in this way and have lost their lives. It is therefore a wise precaution to boil the milk taken by children; indeed, when the source from which it is obtained is not known it is absolutely necessary to do so.

That **consumption can be caught** by the healthy from a consumptive patient is now a well authenticated fact. The tubercle bacilli abound in the expectorations of the consumptive. These should not, therefore, be spat on to the floor or ground and left to dry, for in this way the bacilli are disseminated in the atmosphere, and if then inspired into the lungs they may induce consumption in the nurse or attendant on the sick. Hence it is of the utmost importance that the expectorations of consumptives should be spat into covered vessels, and that they should be carefully collected and burnt, and that other like precautions should be taken in the home and in the sleeping apartment.

### Irish Hospitals.

### THE ROYAL HOSPITAL FOR INCURABLES, DUBLIN.

The Royal Hospital for Incurables at Donnybrook is one of the most popular as well as one of the finest of the hospitals in Dublin. It is beautifully situated in its own extensive grounds, and has a most cheerful aspect. Its inmates number about 180, but with the new pavilion just completed the hospital will comfortably accommodate 230 patients. This pavilion has a southern aspect, and consists of two wards, the upper one being reserved for consumptives only. It has a beautiful bay window at one end, from which the patients have a fine view over the grounds of the hospital, and in the distance the Dublin mountains are visible. The wards all through the hospital are long and lofty, and are arranged in cubicles divided by wooden partitions about 6 ft. high. Each cubicle is furnished according to the owner's taste, as a little bed-sitting-room. Some, in fact most of them, are very pretty and bright. Each has its own window, which is an obvious advantage. In these windows some of the patients keep ferns and flowers.

Every ward contains from twenty-two to twenty-six beds, and in connection with each of the female wards there is a small kitchen. A variety has been effected between the wards by the selection of different colours in the painting of the wood-work; these colours have been chosen for brightness and freshness, and are arranged in a most pleasing manner. The corridors throughout the institution are beautifully kept.

In the last report of the hospital, which was published in March, it appears that 22 patients have been in the institution from 10 to 15 years, 6 from 15 to 20 years, and 16 have remained 20 years and upwards. All the patients seem quite happy and contented, and thoroughly enjoy a chat. They do not, by any means, remain secluded in their respective cubicles, but meet and enjoy each other's society, especially in the beautiful large window which every ward possesses, and which is always a popular resort. They take their needlework there, or the newspaper, and discuss both with their companions.

Visitors to the hospital are numerous, and from time to time ward concerts are given, at which many well-known local artists perform. These concerts are the greatest pleasure and excitement to the patients, and give them plenty to talk and think about for a long time, and they are more frequent in this hospital than in any other in Dublin. Some kind and thoughtful friends have presented a piano to each ward, so that the trouble which exists in many places of carrying a piano from ward to ward is avoided; hence, doubtless, the frequency of concerts and entertainments. There are two nurses to each ward, and these are under the able and kindly management of Miss Stewart, the Lady Superintendent. The resident medical officer is Dr. W. Russell, L.R.C.P.I., L.R.C.S.I.

The hospital is not a palace of idleness to the patients by any means, as many practise their trades if they are such as can be followed without injury to health. Consequently they are much happier than they could possibly be without their usual employments.

There are no religious distinctions made in the hospital, Roman Catholics and Protestants being equally admissible, though some of the wards are exclusively reserved for the former and some for the latter. This is not the case in all, as there are several mixed wards. However, it is intended, as far as possible, in the future to keep them separate, as it is generally found to be more agreeable for the patients themselves.

The corridors are generally considered one of the chief beauties of this hospital. They are long, bright, and airy, and in unsettled or inclement weather they can be used by the patients as a promenade. Of course, when the weather is fine, nothing could be pleasanter for invalids than the grounds, where there are seats arranged with a kind of canvas awning round them, where fresh air and sunshine can be enjoyed without risk. The patients seem thoroughly well looked after, nothing being left undone to make them as happy and comfortable as it is possible for invalids to be.

### Where to Go.

The National Sunday League Musical Society of which Sir Arthur Sullivan is the president, will give oratorio performances at the Queen's Hall, conducted by Mr. C. Sibley, on Sunday evenings. The "Messiah," "Elijah," "Last Judgment," "Creation," "Stabat Mater," "Prodigal Son," "Judas Maccabæus," and Dr. Hubert Parry's "Job" are announced for Sunday concerts during the winter; to be given also at Shoreditch Town Hall; the Horns, Kennington; Bermondsey Town Hall; and Stanley Hall.

### Novelties for Nurses.

Some delightful flannels are supplied by Messrs. Barker and Moody, of Leeds, of a special unshrinkable quality. They are made in a great variety of shades, and it is really difficult to name one's favourite colour in face of so many pretty ones. Anybody wishing for jackets or dressing gowns, shirts or frocks, will be wise to get patterns from this well-known firm.

### Presentation.

Miss Bower, late Matron of the Manchester Hospital for Consumption, Bowden, was presented by the nursing staff with a silver card-case, and by the Secretary with a fern-holder, shaped like a thistle, on the occasion of her departure. The patients gave a polished silver-mounted biscuit barrel, bearing a suitable inscription.

☞ For Structure and Care of the Teeth, Women's Work, Jottings from India, &c., see p. ccxliii. et seq.

### Structure and Care of the Teeth.

### IV.—CHILDREN'S TEETH TROUBLES.

THE question is often asked when should a nurse begin to brush a child's teeth? It is impossible to begin the use of the brush too soon. The first time the child's mouth is washed, let a small, suitable brush, not a sponge, be used at least twice a day, thoroughly and carefully. It is a hopeless task to attempt to persuade boys and girls of from eight to sixteen—a time of life when the will is strong, and neither care for the future nor interest in personal appearance has taken any hold upon the mind—to pay attention to their teeth, if the habit of cleanliness in this respect has not been formed in early childhood.

Let the child grow up from its earliest consciousness with a feeling that a tooth brush is a daily necessity to be faithfully used, and the habit will not be easily set aside when the mind begins to act for itself. See that the teeth are really brushed in a commonsense manner, as a piece of plate would be cleaned for the table. No good housekeeper would allow a piece of plate to be put on the table with all the prominent parts rubbed bright and all the depressions and crevices full of dirt. But that is exactly what almost everybody does with his teeth, and certainly it is what all children will do if they are not taught better.

To do the work properly the brush must be of just sufficient hardness or softness to allow of its being forced well into all the interstices, inside and outside, with an up and down movement as well as backwards and forwards, and transversely across the front, permitting a good, vigorous rubbing of both teeth and gums, but without wounding the latter. When the teeth are not very hardy a thread of very loosely twisted silk or linen, well waxed with common bee's-wax, should be passed through all the interstices, and every fragment of food removed, either in this manner or with a toothpick. Few people realise that a tooth has five sides, and it is curious how often the surfaces next to the tongue and palate are unaccountably neglected.

A perfect toothbrush is difficult to obtain. For adults it is best made of horse-hair, and for young children of the finest hairs, whilst for babies badger hair is hard enough. Any brush will be worthless in a month if always kept wet, since the hairs will rot and come off. It should be carefully dried and hung up, fully exposed to the air, until required again. If treated thus it will last six months at least, which is quite as long as any toothbrush should be used.

We see men and women, in good average health and strength, losing their teeth at forty or fifty years of age, when there can be no question that a reasonable amount of instruction and good management in childhood would have enabled them to save a fairly good and useful set until they reached old age.

Teeth which were delicate in structure break down and crumble away from neglected decay in youth, and those which were strong and hardy become loose and fall away, from disease in the gums, arising from simple ignorance of what a clean mouth means. It is perfectly true that nature has not provided us with a toothbrush, ready made, and to some minds this seems a sufficient reason why we should not use one. But nature also neglected to provide a way of disposing of the refuse which might accumulate about our residences, and it took years of unwearying persistence on the part of the early teachers of sanitary science to convince us that we must do something ourselves to remedy this oversight of Mother Nature.

Scientists have won their battle against ignorance and superstition, and we may hope that in time mothers will learn that cleanliness in the mouth is the only hope for arresting the rapid progress of deterioration, and that it depends on them to learn the lesson well, and teach it to their children.

Sugar is almost as necessary an article of food for the young as bread itself, and it is only an acid return from the stomach, resulting from intemperate use of sugar, that can do any injury to the teeth. It is, of course, well to regulate the supply of sugar to young children, but not to forbid it.

Another absolute fallacy about the teeth is that medicines destroy them. The teeth may, and do, decay, when we are ill and taking medicine, and in some forms of illness the mischief done to the teeth is very serious indeed; but it is the nature of the illness itself which, by vitiating the secretions of the mouth, destroys the teeth.

As soon as a child is able to understand what you mean by rinsing the mouth—as soon as it is capable of holding some water in the mouth for a few seconds and then putting it out—you should begin to use some simple tooth powder. Precipitated chalk is a very good thing to commence with. At three years of age you may safely use a powder which conains some soap as well as chalk, or other delicate gritty substance. There are many soapy tooth powders, and they are nearly all good. Soap is an essential ingredient; so much so, that no tooth powder or dentifrice can be considered worth having unless one-third part of it is soap. It is best to put these preparations in a wide-mouthed stoppered bottle, and from this to put out a week's supply into an ordinary tooth-powder pot for the washstand.

It is a matter of everyday experience to see children of three or four years of age suffering from toothache, which is absolutely preventible if mothers will look after the mouths of their little ones properly. Careful brushing twice a day, and a visit to the dentist when any decay is detected, will certainly prevent this decay from increasing to such an extent as to cause pain, and (what is not often thought of) rendered useless for mastication. Nobody doubts the importance of teeth in the preparation for digestion of the food of the adult, but how very few ever think of how the roll of infant mortality is swollen by ailments arising from indigestion.

The temporary teeth, ten in number in each jaw, will usually have appeared when the child is three years old. A little variation from this does not matter much, nor is it of much consequence to the future of the mouth whether there is regular or irregular arrangement.

A child should never be allowed to contract habits of sucking the thumb, fingers, or lip, or tongue, for all of these interfere with the proper development of the jaw, and often produce serious malformations which are troublesome and difficult to correct. While the bones are still soft it is easy, by continuous pressure, to alter their shape; and when a child is sucking the thumb, the weight of the hand and arm is applied so as to flatten or press in the anterior portion of the lower jaw, and extend the upper, thus producing a protrusion of the upper jaw and teeth. In sucking the fingers the weight of the hand tends to elongate the lower jaw, and the child will have an "underhung" jaw. We have seen the models of two mouths, in which thumb-sucking was permitted until the children were twelve years old, so that probably as much mischief was done as could be done by this habit. In the one case the hand was held in the ordinary position, with the thumb pointing upwards, and the effect was to elongate the upper jaw and check the growth of the lower. In the other case the hand was inverted, the thumb pointing downwards, and, of course, the effect was to pull forward the lower jaw.

Soon after a child has all the temporary teeth, it is perfectly natural and right that separations should begin to appear between them, and for these to increase until all the front teeth have wide spaces between them to make room for the permanent teeth, the crowns of which are rapidly approaching completion at this time, and which are usually about half as wide again as the temporary teeth, and fully grown in width when they first appear through the gums; so they always appear large and out of proportion to the child's face, until the face grows more mature.

We often see these temporary teeth very seriously decayed, and it is always wise to seek professional advice in such a case.

### Women's Work.

### MEDICAL WOMEN.

The number of women who adopt the profession of medicine is steadily increasing, and the well-trodden road they now follow contrasts favourably with the narrow and difficult pathway up which the first female students so courageously struggled. Opposition is everywhere modified, and in some quarters is altogether absent.

Many parents look with equanimity upon a daughter's decision to take to medicine, for they begin to realise the justice of giving their girls and boys equal educational advantages.

But the fees of the medical student (payable in advance) form but a portion of the outlay required. There are books to be purchased, board, lodging, and pocket-money to be supplied during the five years which her medical studies must cover.

Now that so many women workers live "in rooms," the demand has created a supply of accommodation more or less suited to the various tastes of students. There are ordinary boarding houses where punctually served meals are provided, and bed-sitting rooms which form fairly quiet studies, whilst the residents are spared all housekeeping anxieties. Again, there are houses for students only, as well as ordinary furnished lodgings in which the inmate has to provide for herself. A popular plan is for three or four students to take a small house between them and share expenses, doubtless a comfortable and homelike mode of living, but a good housekeeper is essential to make the scheme work smoothly.

The student's school life is mapped out definitely for her, and she can take the various examinations rapidly or slowly as she pleases. The young girl fresh from school rushes straight through the course which means to her merely a continuance of her former arduous labours, and it is, therefore, small wonder that the added responsibilities fail at first to impress her. A woman beginning her studies at a somewhat later date often takes the examinations more deliberately, partly because they come harder on her than on the school girl, and partly on account of her wider views of life and deeper sense of the serious after-work for which the medical school is merely the preparation.

When the course is completed and the compulsory hospital attendances "signed-up," the duly qualified woman has to decide "what next?"

If her taste lies in the direction of an Indian career she has little trouble in gratifying it, for competent women are in great demand, and the work in the vast empire is well-nigh limitless. Most of the aid required by the women of India can only reach them by means of their own sex, and, therefore, lady doctors have an enormous field of usefulness where no competition is to be feared from the other sex.

If taste or family ties make going abroad undesirable, the newly-qualified doctor has to try for a post at one of the few hospitals or kindred institutions where women are permitted to practice. A few general practitioners also take assistants in country districts, and experience is thus attainable, though the remuneration given is sometimes far less than would be offered to a man holding similar qualifications.

Thus it is still difficult for a woman to secure even a small portion of the valuable experience which falls to the lot of the qualified man, even women's hospitals, which seem the natural home of the lady doctor, being not infrequently still closed to her.

Yet, now that so many advantages have been obtained for medical women by the persistent energy of their predecessors, it is only natural to foresee that opportunities of gaining practical as well as special experience will not long be denied them.

### A Case for Assistance.

We rejoice to be able to state that the total sum subscribed by our readers now amounts to £30 11s., so that it will be possible to provide for the old matron in distress adequately and well. We are most grateful to our readers for the generosity they have displayed, and would especially thank the matrons, sisters, and nurses who have combined in so many hospitals to raise a special subscription for this case. Contributions received up to date: H. D., Kensington, 20s.; D. H., London, 5s.; Nurses at Weston-super-Mare, 9s. 6d.; Matron and Nurses, Cancer Hospital, London, 20s.; making a total of £30 11s.

### Appointments.

Lewisham Infirmary.—Miss Marie Campbell has been appointed Assistant Matron at this infirmary. She was trained at the Royal Hants County Hospital, Winchester. Appointed Night Superintendent of the Royal Infirmary, Southampton, Miss Campbell subsequently became Sister-in-Charge of the male medical and then of the male surgical ward and operation theatre there. She has been working at Southampton for four years, and has won golden opinions from everybody. Miss Campbell is a born administrator, as we have testified already in these columns, and her knowledge, capacity, and character mark her out for high office and responsibility.

Liverpool Hahnemann Hospital and Homœopathic Dispensaries.—Miss M. Bower has been made Lady Superintendent of this institution. She was trained at the General Infirmary, Derby, and the London Hospital, Whitechapel, and worked for nearly three years at the General Infirmary, Gloucester. Miss Bower then became Matron of the Manchester Hospital for Consumption at Bowden, and takes many good wishes with he in her new work.

### Minor Appointments.

Hope Hospital, Eccles.—Miss Maggie Morrison has been made a charge nurse at this hospital at the expiration of her training. She previously worked for two years at the King's Cross Fever Hospital, Dundee. Miss Annie G. Baker has also been made a charge nurse at the Hope Hospital, where she received her training. Miss Emma Musker, also trained and certificated at this hospital, has been made nurse at the City Hospital, Grafton Street, Liverpool. We wish all three nurses every success, and congratulate them on their satisfactory certificates.

### Notes and Queries.

### Queries.

(143) *Training.*—Is there a hospital where anyone aged 35, with a certificate for monthly nursing, can get a year or two's general training without a premium?—*E. A. B.*

(144) *Badges.*—Are any special badges worn by the nurses at Samaritan Hospital?—*Constant Reader.*

(145) *Training.*—Can you recommend me where to apply for training for a young probationer for three months in midwifery and general nursing?—*C. S.*

(146) *Inspection.*—Where can I get information as to examination for inspectors of nuisances?—*J. G.*

### Answers.

(143) *Training (E. A. B.).*—We fear your age is above that at which general probationers are admitted to London hospitals. You might write to some of the large provincial infirmaries where the matrons are themselves trained, and ask for a copy of the rules.

(144) *Badges (Constant Reader).*—You had better address your query to the matron of the hospital in question.

(145) *Training (C. S.).*—We advise you to let the young woman go into a really good hospital for three years, and then give her three months special training in midwifery. No one can be "trained" in general nursing in three months. The word is misapplied in such a connection.

(146) *Inspection (J.G.)* Apply to secretary, Sanitary Institute of Great Britain, Parkes Museum, Margaret Street, London.

### Jottings from India.

By a Correspondent.

Last year the Presidency of Bengal suffered less than usual from cholera, there were scarcely any cases among British troops, and the mortality from cholera among Europeans was small. This year it has broken out, and even the hill stations have not escaped. Allahabad has been one of the seats of the outbreak; one artilleryman and one of the 87th have died, also the wife of one of the medical officers. It first appeared at Lucknow amongst the East Lancashire Regiment, their barracks being close to the Sudder Bazaar. Some of the 18th Royal Irish have also succumbed. Up to present date 140 cases have broken out in the cholera camp at Lucknow, and there have been 103 deaths, amongst them the Quartermaster of the East Lancashire Regiment. The officers have all been recalled from leave to rejoin at Lucknow. Very little oozed out into the public papers, only such details as: "At Lucknow on Friday night an electric storm raged most furiously, attended by a perfect deluge of rain. No better tidings are forthcoming from the cholera camp over the Gumti, where the East Lancashires have the stress of weather added to their other afflictions"; and "Owing to an outbreak of cholera, the Gorakhupur Jail has had to be hastily cleared of its occupants. There are now some 700 convicts out in camp." At Cawnpore, two hours' journey from Lucknow, the troops are "cholera dodging," and out in camp. Some of the Munster Fusiliers have died. "Considerable showers of rain at Cawnpore; the health of the station is better in consequence. Very few fresh cases of cholera have been reported, and some almost hopeless cases have even recovered; more than 75 per cent. of the victims were Mahammadans. Mr. Hankin, a pupil of Professor Haffkine's, and Government analyst, is at present at Cawnpore on a tour of inspection. The wells, particularly in the Civil Lines, have been found for the most part plentifully stocked with the ubiquitous microbe. Of eighteen wells examined last week thirteen were found impure. One rather extraordinary thing in connection with the cholera epidemic at Cawnpore has been the rush for ice on the part of every caste in the native community. No sooner is an individual attacked than his friends get it for him at any price. Dealers in the commodity have consequently reaped a golden harvest, often getting as much as 4 annas to 6 annas per seer." (A seer is equivalent to 2 lbs. English weight.) There has been some cholera at Dinapore, and General Evans, C.B., commanding the Allahabad district, went down on a recent Saturday to inspect the arrangements. The cholera epidemic is increasing in Mandalay. While cholera is raging down South, it is also bad in Bombay. The other night it broke out suddenly in one of the wards of Jamsetjee Jeejeebhoy Hospital. The next news was of forty deaths. At present "the condition of affairs at the Jamsetjee Jeejeebhoy Hospital is much improved, only nine cholera patients being under treatment, and these are progressing favourably. All the wards have been cleansed and disinfected, and everything is being done to prevent a fresh outbreak. There are only about 20 patients lying in the hospital, which can accommodate 400. The drainage of the hospital premises is supposed to have caused the recent outbreak."

### The Book World for Women and Nurses.

[We invite Correspondence, Criticism, Enquiries, and Notes on Books likely to interest Women and Nurses. Address, Editor, The Hospital (Nurses' Book World), 428, Strand, W.C.]

In the Dwellings of Silence: A Romance of Russia. By Walter Kennedy (London: Heinemann, 1894. 1 vol.)

So long as the Russian Czar, the "Bogie Man" of the nineteenth century, banishes his political prisoners of both sexes to Siberian horrors, so long will English readers welcome romantic and quite impossible stories of escapes across the deserts of snow. There is nothing particularly new in the "Dwellings of Silence," but although the subject is an old friend, the writing is fresh and vigorous, and the adventures are described so vividly as to almost set incredulity at defiance. The tale opens with a ball at the Winter Palace at St. Petersburg. The belle of the ball is the heroine of the story, Valérie Melnikoff, a beautiful Nihilist who receives two proposals of marriage during the evening. Ivan Valerianoff, a young Russian Hussar, she rejects, but Frank Devereux, an American Secretary of Legation, she accepts, thereby incurring the bitter resentment of Ivan. When Devereux assures her that his love for her is so deep that he would follow her "even to Siberia," the least astute reader at once realizes that he will be called upon to fulfil his rash promise. The next day sees Valérie betrayed to the police by Ivan and incarcerated with other Nihilists of equally innocent lives in the gloomy fortress by the river. Then comes the weary march to Siberia, and the reader is spared no details of the horrors of the way, which result in the death of one of the women prisoners. The history of the escape of Valérie and her friends from the prison, planned and executed by Devereux disguised as a prison doctor, is very well worked out, and the local descriptions are admirable. The idea of chloroforming all the prison authorities and sentries is rather incredible, but as the exciting escape through the snow could not have been effected without the successful accomplishment of this astonishing feat, one must not quarrel with it, although it raises the question whether truth be stranger than fiction. Ivan is the person to arouse the sleeping prison and give the alarm, and he follows the fugitives himself, who baffle him at every turn. After many weary months the exiles arrive at the coast, having outdone Robinson Crusoe in the ingenuity of the way they supported life under difficulties. A friendly boat conveys them to the land of the Star-spangled banner, where Valérie bestows her well-earned hand on Frank Devereux.

### Books Received.

Young J. Pentland.

"The Treatment of Wounds, Ulcers, and Abscesses." By W. Watson Cheyne, F.R.S.

J. B. Lippincott and Co. (Philadelphia).

"International Clinics." Vols. I. and II. Fourth series.

T. Nelson and Sons.

"Corea of To-day," Price 6d.

Longmans, Green, and Co.

"Hygiene." By J. Lane Notter, M.A., M.D., and R. H. Firth, F.R.C.S.

Simpkin, Marshall, Hamilton, Kent, and Co.

"Myxœdema, Cretinism, and the Goitres." By Edward P. Blake, M.D., M.R.C.S.

Messrs. Oliver and Boyd.

"The Insanity of over-exertion of the Brain." By J. Batty Tuke, M.D.

Messrs. Eyre and Spottiswoode.

"The Sale of Food and Drugs: the Acts of 1875 and 1879." By T. C. Hedderwick, M.A.

Bailliere, Tindall, and Cox.

"Table of Doses and Strengths of the British Pharmacopœia." Price 6d.

Kegan Paul, Trench, Trubner, and Co. (Limited).

"The Jewish Method of Slaughter, Compared with other Methods from the Humanitarian, Hygienic, and Economic Points of View." By J. A. Dembo, M.D.

**Periodicals and Pamphlets.**—Cornhill, English Illustrated Magazine, St. Nicholas, Cassell's Family Magazine, Sunday at Home, Leisure Hour, Indian Engineering, Amateur Gardening, Reich's Medicinal Anzeiger, Service for the King, Child's Guardian, Humanitarian, The Planter, Girls' Own Paper, Boys' Own Paper, Invention, Rural World, Public Opinion, Journal d'Hygiène, Publishers' Circular, Westminster Review, Public Health, Maanblad voor Ziekenverpleging, Revue Générale des Sciences, Monthly Homœopathic Review, Provincial Medical Journal, L'Ichthyol dans le Traitement des Uréthrites et des Cystites, &c., Monatshefte für Praktische Dermatologie, London, Children's League of Pity Paper, Journal of Education, Toilers of the Deep, The News, Health Record, Tellustria (by B. G. Jenkins, F.R.A.S.), Christian World, Friendly Greetings, Our Little Dots, Child's Companion, Light in the Home, Cottager and Artisan, Illustrated Penny Tales, Tit Bits, The Million, Picture Magazine, and Strand Magazine.

## Figures and Tables

**Fig. 1. f1:**